# Real-World Evidence Synthesis of Digital Scribes Using Ambient Listening and Generative Artificial Intelligence for Clinician Documentation Workflows: Rapid Review

**DOI:** 10.2196/76743

**Published:** 2025-10-10

**Authors:** Naga Sasidhar Kanaparthy, Yenny Villuendas-Rey, Tolulope Bakare, Zihan Diao, Mark Iscoe, Andrew Loza, Donald Wright, Conrad Safranek, Isaac V Faustino, Alexandria Brackett, Edward R Melnick, R Andrew Taylor

**Affiliations:** 1 Department of Biomedical Informatics and Data Science, Yale School of Medicine, New Haven, CT, United States; 2 VA Connecticut Healthcare System, US Department of Veterans Affairs, West Haven, CT, United States; 3 Department of Emergency Medicine, Yale School of Medicine, 464 Congress Avenue, #260, New Haven, CT, 06519, United States, 1 203-737-7694; 4 Centro de Innovación y Desarrollo Tecnológico en Cómputo CIDETEC, Instituto Politécnico Nacional, Mexico City, Mexico; 5 Department of Biostatistics (Health Informatics Division), Yale School of Public Health, New Haven, CT, United States; 6 Harvey Cushing/John Hay Whitney Medical Library, Yale University, New Haven, CT, United States; 7 Department of Emergency Medicine, University of Virginia, Charlottesville, VA, United States

**Keywords:** digital scribes, artificial intelligence in medicine, clinical documentation, speech recognition software, patient-clinician communication

## Abstract

**Background:**

As physicians spend up to twice as much time on electronic health record tasks as on direct patient care, digital scribes have emerged as a promising solution to restore patient-clinician communication and reduce documentation burden—making it essential to study their real-world impact on clinical workflows, efficiency, and satisfaction.

**Objective:**

This study aimed to synthesize evidence on clinician efficiency, user satisfaction, quality, and practical barriers associated with the use of digital scribes using ambient listening and generative artificial intelligence (AI) in real-world clinical settings.

**Methods:**

A rapid review was conducted to evaluate the real-world evidence of digital scribes using ambient listening and generative AI in clinical practice from 2014 to 2024. Data were collected from Ovid MEDLINE, Embase, Web of Science–Core Collection, Cochrane CENTRAL and Reviews, and PubMed Central. Predefined eligibility criteria focused on studies addressing clinical implementation, excluding those centered solely on technical development or model validation. The findings of each study were synthesized and analyzed through the QUEST human evaluation framework for quality and safety and the Systems Engineering Initiative for Patient Safety (SEIPS) 3.0 model to assess integration into clinicians’ workflows and experience.

**Results:**

Of the 1450 studies identified, 6 met the inclusion criteria. These studies included an observational study, a case report, a peer-matched cohort study, and survey-based assessments conducted across academic health systems, community settings, and outpatient practices. The major themes noted were as follows: (1) they decreased self-reported documentation times, with associated increased length of notes; (2) physician burnout measured using standardized scales was unaffected, but physician engagement improved; (3) physician productivity, assessed via billing metrics, was unchanged; and (4) the studies fell short when compared to standardized frameworks.

**Conclusions:**

Digital scribes show promise in reducing documentation burden and enhancing clinician satisfaction, thereby supporting workflow efficiency. However, the currently available evidence is sparse. Future real-world, multifaceted studies are needed before AI scribes can be recommended unequivocally.

## Introduction

Health care has arguably failed to preserve one of the fundamental pillars of medicine: protecting time for meaningful patient-clinician communication. For each hour spent on direct patient care, physicians now spend up to 2 hours on the electronic health record (EHR), both during and outside clinic hours [1,2]. This shift toward increased documentation and EHR-driven tasks, driven by billing, medicolegal, and regulatory requirements, has led to more time in front of the computer and strained the patient-physician relationship [1,3,4]. The effects have been far-reaching, impacting patient care quality and contributing to physician burnout [3,5-8].

To address these challenges, health care has turned to digital tools promising to offload work burden, revive patient-clinician interactions, and reduce burnout [9]. In particular, digital scribe technology, spurred on by recent advancements in automated speech recognition, increased computational power, and breakthroughs in the development of large language models, which underlie services such as ChatGPT, has enabled rapid advancements in automated documentation [10-12]. These digital scribes, which use a listening device (such as smartphones) to ambiently record patient-clinician conversations and automate generation of a suitably formatted clinical note (eg, a structured progress note) on completion of the encounter, present a potentially promising solution to the pernicious problem of documentation burden. And, despite the relative novelty of digital scribe technology (Nuance DAX, 2020 being the first major tool in the market [13]), and perhaps because of the pressing issues it aims to address, numerous next-generation digital scribes have already entered the market and clinical practice.

Despite the rapid implementation of digital scribes, real-world research on their effectiveness in improving documentation quality, patient safety, outcomes, and physician well-being remains limited [14]. Medicine and health IT are replete with examples of prematurely adopted technologies that failed to meet expectations [15]. Robotic surgical systems, for instance, were widely embraced with promises of precision and faster recovery, but subsequent studies revealed mixed results, highlighting limited benefits over traditional surgery and raising concerns about high costs and steep learning curves [16]. Similarly, wearable health monitors such as fitness trackers and smartwatches, initially praised for enhancing patient engagement, often produce data that are not clinically actionable and can contribute to patient anxiety, without clear evidence of improved health outcomes [17,18].

Given the rapid adoption of these tools in clinical practice, there is a pressing need to evaluate their effectiveness in real-world settings [4,14,19]. While many studies have examined algorithmic performance, assessments of how these technologies influence routine care, including everyday clinical workflows, physician satisfaction, and patient care quality, are just emerging. This rapid review seeks to address this gap by synthesizing the current literature on the practical use of artificial intelligence (AI)–enabled digital scribes in clinical environments.

## Methods

### Study Design and Rationale

We applied a rapid review approach to assess the real-world impact of quickly evolving digital scribe technology [20]. Unlike systematic reviews, which are comprehensive but time intensive, a rapid review provides timely, relevant findings to support immediate decision-making [21]. Compared to scoping reviews, which broadly map research areas, our rapid review focuses on synthesizing actionable evidence on the effectiveness of digital scribes [22]. This approach balances rigor and timeliness, making it well-suited for evaluating evolving technologies.

### Literature Search—Selection Criteria and Search Strategy

We searched Ovid MEDLINE, Embase, Web of Science–Core Collection, Cochrane CENTRAL & Reviews, and PubMed Central to discover relevant articles. The search strategy was developed in conjunction with a professional librarian to ensure comprehensive coverage of the topic. The strategy used a broad approach that included AI concepts implied in the metadata, thereby capturing articles that might otherwise be excluded by discrete AI-related terms.

We included papers that mentioned ambient listening technology within the health care domain within the last 10 years (2014‐2024), particularly following the advent of AI and advanced transcription technologies, with a focus on practical impact on physicians and staff, rather than theoretical model development. We excluded studies focused solely on model development, nonclinician populations, and technologies other than ambient listening using generative AI. In addition, studies outside the specified time frame, theoretical papers, and those not in English were excluded. The full search strategy is available in Multimedia Appendix 1.

### Data Extraction

To ensure uniformity and consistency in data extraction, a standardized data abstraction tool, subsequently referred to as the evidentiary table, was developed collaboratively and agreed upon by all authors. Articles that met the predefined inclusion criteria were assigned equally among team members for independent review. Each article was reviewed by at least 2 reviewers, with a third reviewer available to adjudicate any discrepancies. The review team included NSK, YVR, TB, ZD, IVF, and CS. Extracted data were systematically recorded using an Excel (Microsoft Corporation) spreadsheet to create an evidentiary table for synthesis.

The review process followed a systematic, multistage approach corresponding to the identification, screening, and inclusion stages specified in the PRISMA (Preferred Reporting Items for Systematic Reviews and Meta-Analyses) guidelines. In the title and abstract screening phase, studies were screened for eligibility, with discrepancies among reviewers being documented, and interrater reliability measured using prevalence-adjusted bias-adjusted κ (PABAK) and AC1 statistics. Following this phase, selected articles proceeded to the full-text review, where they were further evaluated against predefined inclusion criteria. This stage also included interrater reliability measures to assess reviewer agreement, ensuring consistency.

### Data Synthesis and Organization

The data synthesis for this study was conducted using a structured thematic approach guided by the QUEST human evaluation framework [23], Systems Engineering Initiative for Patient Safety (SEIPS) 3.0 model [24,25], and a study by Abbasian et al [26]. These frameworks were used to ensure that the data extraction and organization comprehensively addressed all relevant aspects of digital scribe implementation, including clinician efficiency, satisfaction, safety, quality, and practical barriers in real-world clinical settings.

We used the QUEST Human Evaluation Framework to assess the quality and safety of information provided by digital scribes [23]. Specifically designed for evaluating LLMs in health care, QUEST comprises 3 phases: planning, implementation and adjudication, and scoring and review. It is guided by 5 key principles: quality of information, understanding and reasoning, expression style and persona, safety and harm, and trust and confidence. In this study, QUEST was used to evaluate generated clinical documentation for accuracy, bias, comprehensiveness, and safety issues, such as transcription errors and hallucinations.

The SEIPS 3.0 model was applied to evaluate the broader impact of digital scribes on patient safety and health care processes. SEIPS 3.0 focuses on the patient journey and how health care activities are distributed across different settings and times. This framework guided assessment on how digital scribes influenced patient outcomes (eg, engagement and satisfaction), clinician outcomes (eg, efficiency, workload, burnout), and organizational outcomes (eg, workflow and productivity). We used the study by Abbasian et al [26] as a basis to evaluate foundation metrics of user perspectives and real-world contexts. In their paper, they describe the health care metrics in 4 domains, namely accuracy, trustworthiness, empathy, and performance.

### Reviewer Assessment

To maintain accuracy in the selection and abstraction process, both title and abstract screening and full-text review phases included measures of interrater reliability. We used 2 specific metrics: PABAK and Gwet AC1 [27]. These measures were selected due to their robustness in handling data with a high prevalence of rater bias, common in systematic reviews where agreement on inclusion criteria can be challenging. PABAK was chosen as it adjusts for both prevalence and bias, providing a more stable agreement measure when the likelihood of certain ratings is high, thus avoiding the common limitations of Cohen κ in such scenarios. Gwet AC1 was included because it similarly accounts for prevalence effects but is less sensitive to rater bias, making it an optimal complement to PABAK for accurately capturing agreement levels. Both metrics offer a reliable, comprehensive view of interrater reliability, aligning with our study’s emphasis on precision and consistency in data extraction [28,29].

## Results

We begin by outlining the key characteristics of the review process, followed by the main study results categorized by major findings; due to the diversity of study types and outcomes, the results were not combined. Table 1 summarizes each study’s title, aim, and key outcomes.

**Table 1.** General characteristics of the studies included in this rapid review.

Study	Authors and year	Objective	Sample size	Study design	Setting	Tool/ vendor	Key findings
AI^a^-driven digital scribes in clinical documentation: pilot study assessing the impact on dermatologist workflow and patient encounters	Cao et al [30], 2024	Explore the use of DAX^b^ as a digital scribe in an academic and community-based dermatology setting	12 dermatologists	Research letter	Not available	Nuance DAX	Digital scribes decrease average documentation time by 22% (90.1-70.3 min/d), ease administrative burdens, and improve both clinician and patient experience in dermatology clinics. A total of 83.3% would be “very disappointed” if the tool was taken away.
Impact of an AI-based solution on clinicians’ clinical documentation experience: initial findings using ambient listening technology	Galloway et al [31], 2024	Report the impact of a pilot implementation of ambient listening on clinicians’ documentation experience in the EHR^c^ and on overall well-being	31 physicians	Survey	Academic Medical Center	Abridge	Positive responses on documentation meeting requirements rose from 41.9% to 71% post implementation, and ease of use improved from 32.3% to 48.4% post implementation. Negative impacts on well-being and patient experience dropped significantly, with 35.5% recommending the solution and 58.1% noting increased productivity. Using DAX over an in-person scribe could equate to US $13,400 to US $14,400 in cost-savings
The impact of nuance DAX ambient listening AI documentation: a cohort study	Haberle et al [32], 2024	To assess the impact of the use of DAX on caregiver engagement, time spent on EHR, productivity, attributed panel size for value-based care providers, documentation timeliness, and CPT^d^ submissions	99 clinicians (DAX users)+76 matched controls	Peer-matched controlled cohort study	Medical Group 12 specialties	DAX	Nuance DAX appears to have no benefit in productivity for fee-for-service clinicians and no improvement in total panel size for value-based primary care. Positive trends in provider engagement were noted, while nonparticipants saw worsening engagement
Implementing digital scribes to reduce EHR documentation burden among cancer care clinicians: a mixed-methods pilot study	Nguyen et al [33], 2023	Assess digital scribe’s feasibility, acceptability, appropriateness, usability, and its preliminary association on clinician well-being	21 clinicians, (paired survey responses from 9 clinicians only)	Mixed methods study	Live clinic settings at a National Cancer Institute	DAX	Among the 9 clinicians who completed the paired survey, perceived sufficiency of documentation time (on a 5-point Likert scale) significantly improved from 2.1 to 3.6 (*P*=.005), but no significant changes were seen in burnout score (3.6-3.9, *P*=.08).
The association between use of ambient voice technology documentation during primary care patient encounters, documentation burden, and clinician burnout	Owens et al [34], 2024	Evaluate the association between ambient voice technology, coupled with natural language processing and AI on primary care provider documentation burden and burnout	110 clinicians	Survey and observation	Not available	DAX	Among high DAX use providers, documentation times decreased by 28.8% per encounter translating to time savings of about 50 min daily. High DAX users showed significantly less burnout compared to the lower use physicians on the OLBI^e^ disengagement subscore
Ambient AI scribes to alleviate the burden of clinical documentation	Tierney et al [11], 2024	Understanding how ambient AI scribes reduce documentation burden, enhance physician-patient encounters, and augment clinicians’ capabilities	3442 physicians, 303,266 patient encounters	Commentary	21 medical centers in primary care, pediatric, hospitalist, mental health, surgical, ED^f^ clinicians	Not declared specifically	Compared to the clinicians not using AI scribe, statistically significant reduction in “pajama time” by 2.5 units and time spent in notes during appointments by 0.5 min. Patient feedback (from a survey of 21 patients) was also positive, with 71% noting increased time spent speaking with their physician and 81% observing their physicians spent less time looking at screens during consultations

a AI: artificial intelligence.

b DAX: Dragon Ambient Experience.

c EHR: electronic health record.

d CPT: Current Procedural Terminology.

e OLBI: Oldenburg Burnout Inventory.

f ED: emergency department.

### Characteristics of the Review Process

#### Number of Papers Identified

**Figure 1.**
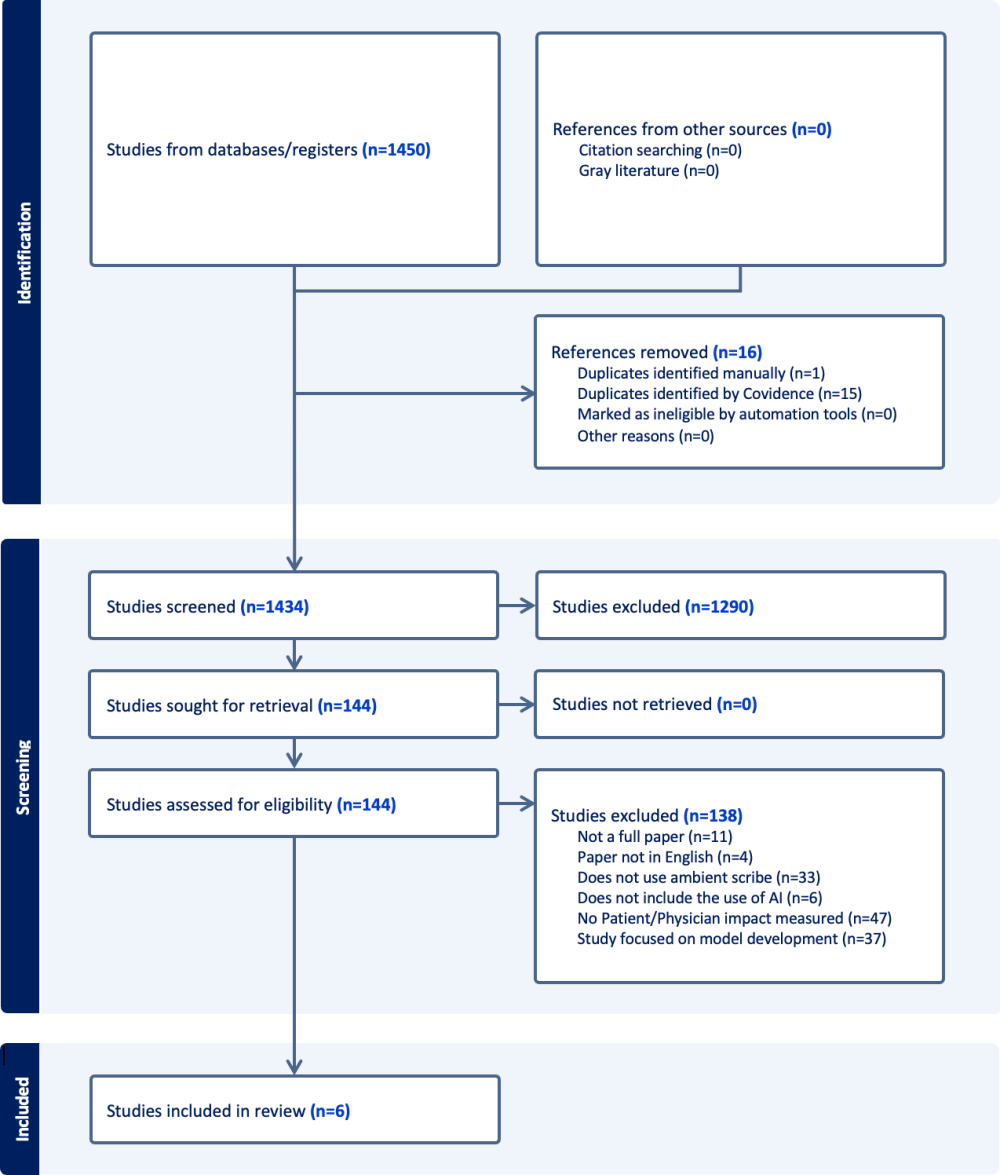
PRISMA (Preferred Reporting Items for Systematic Reviews and Meta-Analyses) diagram.

Out of the 1450 studies identified through database searches, 16 references were removed for various reasons, including duplicate entries (Figure 1). This resulted in 1434 studies being screened. Of these, 1290 studies were excluded during the screening phase, primarily for not meeting the eligibility requirements. Subsequently, 144 studies were retrieved and assessed for eligibility, and 138 were excluded at this stage due to reasons such as the study not being a full paper (n=11), being published in a language other than English (n=4), not involving the use of ambient scribe technology (n=33), not including AI use (n=6), lacking measurements on patient or physician impact (n=47), or focusing solely on model development (n=37). Ultimately, only 6 studies satisfied all inclusion and exclusion criteria and were included in the review (Table 1).

#### Studies That Fit Inclusion Criteria but Were Ultimately Excluded

Several studies initially appeared to meet the inclusion criteria; however, upon detailed review, they were excluded. For example, the paper “AutoScribe: extracting clinically pertinent information from patient-clinician dialogues” is about an ambient AI product, AutoScribe, which has been widely used in some health environments [35]. However, this paper only presents a proof of concept outside a hospital environment without real physicians. Similarly, the paper “A patient-centered digital scribe for automatic medical documentation” presents an ambient AI tool but was tested with medical students posing as patients and not in a real patient-doctor setting [36].

#### Interrater Reliability

In the title and abstract screening phase, 64 discrepancies were identified, with a PABAK of 0.9107 and AC1 of 0.9497, indicating high agreement among reviewers. During the full-text review phase, 13 discrepancies were identified, with PABAK of 0.8194 and AC1 of 0.8927, also demonstrating substantial interrater reliability.

#### Key Findings

Study designs included an observational study, a research letter, a case report, a peer-matched cohort study, 2 survey-based studies, and 1 mixed methods longitudinal study. Sample sizes varied significantly: 3 of them below 30, 2 of them around 100, and one of them 3442 users. The settings were diverse, spanning academic and community health systems and an outpatient dermatology clinic (Table 1).

#### Documentation

##### Documentation Time

Of the 6 studies, 4 studies discussed documentation time. In a pre-post study, among the 19 clinicians who used Dragon Ambient Experience (DAX) in >60% of their visits (“high users”), there was a reported 28.8% lower documentation time per encounter, translating to an average of 1.8 minutes per visit [34]. In a group of 3442 physicians, they observed that time spent in notes decreased from a mean of 5.3 to 4.8 minutes per encounter for AI scribe users, comparable to a reduction from 5.0 to 4.7 minutes for nonusers in the same setting [11]. In addition, the use of ambient AI technology was associated with a 22% decrease in clinical documentation time (90.1-70.3 min/d) in a group of 12 dermatologists [30]. In the fourth study, a subjective ease of documentation process was measured, and 32.3% of the respondents had a positive experience compared to 48.4% postimplementation (*P*=.02) [31].

##### Documentation Length

Documentation length was reported in 2 studies. In a group of dermatologists, compared to manually written notes, the tool-assisted notes showed an increase in length, with word counts rising by 30 to 50 words per note in the machine-transcribed versions [30]. In the same study, the portion contributed by the user decreased by “nearly 50%.” Similarly, in the group of 19 clinicians who used DAX in >60% of their visits (a high-use subgroup drawn from 110 surveyed primary care providers), they reported an increase in documentation length by 542 characters [34].

##### Documentation Accuracy

Among these 6 papers, only 1 study was an explicit attempt to assess the accuracy of notes. They modified the Physician Documentation Quality Instrument and added attributes to measure hallucinations, burden, and bias. In the transcribed notes, they reported an average accuracy score of 48 out of 50 [11]. Furthermore, they reviewed a random sample of 35 notes and noted “few” instances (not quantified) of hallucinations and gave some specific examples in the paper.

### Physician Well-Being

All studies have examined the impact of AI scribes on physician burden in some form, each targeting different aspects of clinician experience.

#### Burnout

Two of the studies addressed burnout. Different standardized tools were implemented to study the burnout in physicians: Mini-Z was used in a group in the cancer center, and they noted nonsignificant changes in the composite scores (33.3 vs 35.9; *P*=.26) [33]. There was significant improvement in the perceived sufficiency of documentation time, which increased from 2.1 to 3.6 on a 5-point Likert scale (*P*=.005) [33]. In a primary care cohort using the Oldenburg Burnout Inventory (OLBI), no significant change was observed overall; however, a high-use subgroup (DAX in >60% of encounters) had a significantly lower OLBI disengagement subscore than lower-use clinicians (Δ=−2.1; 95% CI −3.8 to −0.4) [34].

#### Well-Being

In a study of a multispecialty group of 100 providers, they reported results from the Press Ganey Survey, which showed improvements in the engagement (3.62 vs 3.37), safety (4.16 vs 3.92), resilience/decompression (2.83 vs 2.81), and work-life balance (3.14 vs 2.90) [32]. In a survey-based study, respondents reported an improvement in well-being related to documentation, increasing from 38.7% to 71% (*P*=.01) [31].

#### Pajama Time

“Pajama time,” wherein a physician works after scheduled hours to complete their clinical work, is a well-cited marker of physician burden [37]. This was reported in 2 studies; the mean percent time spent after hours decreased from 14.2% compared to the control group of 14.9% in 1 study [32], and the other reported a “large decrease” (without quantification) [11].

#### Patient Satisfaction

A Press Ganey Survey item, “Likelihood to Recommend,” was used as a measure for patient satisfaction among 99 users by using this tool, but they did not find a statistical difference (86.3% in users to 86.1% in controls) [32]. In another survey-based study, respondents were less likely to report that their documentation process negatively affected the patient experience after implementation of an AI scribe than before the intervention (6.5% compared to 35.5%; *P*=.005) [31].

#### Anecdotes

Three papers reported anecdotes from the users. A mixed methods study reported comments from physicians such as “I can reconnect with the patient” and an overall positive perception and noted it was “acceptable, appropriate, and usable” [33]. The group of dermatologists felt that they would be “‘very disappointed’ if AI scribes were no longer available” [30], and similarly, in the study of 3442 physicians, there were quotes like “this technology was a game-changer” [11]. In the last study, the authors shared the sentiment of “we recognize that the underlying technology and concomitant workflow would continue to evolve rapidly” and “a major goal is to directly integrate artificial intelligence scribe tools into the electronic health record.”

### Barriers

In this commentary by Tierney et al [11], they described some barriers to implementation. One of the barriers mentioned was the reliance on English-only transcription. Although the tool had multilingual support, regulations about certified medical interpretation services in their institution limited its use. In the same paper, they also reported a lack of direct integration with EMRs as a hindrance. In a mixed methods study, they discussed the need for editing, which ranged from minor to major, but reported that eventually, this workload improved as time progressed [33].

### Productivity and Costs of Implementation

Three of the 6 studies discussed associated costs. When examining the effect of DAX in a peer-matched controlled cohort study of physicians across 12 specialties, there was no significant change in the projected work relative value units for the year (ranging from 90.6% to 91.6%) or in Current Procedural Terminology code submission rates (*P*=.57 and *P*=.51), showing no benefit in productivity for fee-for-service clinicians [32]. They corroborated these findings with an internal audit. However, in a convenience sample survey of 117 physicians who piloted an AI scribe at an academic medical center, 58.1% agreed that the tool increased their productivity [31].

The cost of implementation was reported in 1 study with initial setup costs, which ranged from US $1000 for clinician onboarding to US $1850 per month for software use, compared to US $3050 per month for human scribes [30].

### Net Promoter Score

Galloway et al [31] in their survey-based study reported that 35.5% of participants responded that they would be highly likely to recommend this documentation solution to a colleague.

### QUEST, SEIPS 3.0, and Extrinsic Evaluation Metrics

The QUEST human evaluation framework helps one evaluate on the basis of quality of information, bias, and any fabrication, and the SEIPS 3.0 model allows us to study the sociotechnical systems approaches, whereas the study by Abbasian et al [26] allows us to review the proposed intrinsic and extrinsic measures. Tables2  3 describe the comparative analysis of the studies based on these tools [23,25,26].

**Table 2.** Evaluation of included studies using QUEST, Systems Engineering Initiative for Patient Safety (SEIPS) 3.0, and evaluation metrics proposed by Abbasian et al’s [26] frameworks.

Framework, dimensions, and components			Paper		
			Cao et al [30]	Galloway et al [31]	Harbele et al [32]
Health care evaluation metric groups from Abbasian et al [26]					
Accuracy					
Intrinsic, SSI^a^, robustness, generalization, conciseness, up-to-dateness, groundedness			Not considered	Not considered	Not considered
Trustworthiness					
Safety and security, privacy, bias, interpretability			Not considered	Not considered	Not considered
Empathy					
Emotional support, health literacy, fairness, personalization			Not considered	Not considered	Not considered
Performance					
Memory efficiency, FLOP^b^, token limit, number of parameters			Not considered	Not considered	Not considered
QUEST human evaluation framework					
Quality of information					
Accuracy			Not considered	Not considered	Internal audit: no meaningful evidence of “overcoding, missed risk-adjustment opportunity, or insufficient supporting documentation.”
Safety and harm					
Bias			Not considered	Not considered	Not considered
Fabrication, falsification, or plagiarism			Not considered	Not considered	Not considered
SEIPS 3.0 Model					
Other outcomes for patients					
Physical, mental, and emotional health			Not considered	Not considered	Not considered
Efficiency and effectiveness of care			Not considered	Not considered	Internal audit: No meaningful evidence of “overcoding, missed risk-adjustment opportunity, or insufficient supporting documentation.”
Patient experience and satisfaction			78.3% considered that the provider spent less time in the computer, 73.9% considered that the visits felt more like a personable conversation, and 47.7% stated that the provider seemed to be more focused on me during the visit	Significant improvement of perceived patient experience but still low values on a Likert scale (averaged of 1.0/5.0)	Monthly “likelihood to recommend” reports statistical differences in satisfaction (86.3% vs 86.1%)
Other outcomes for clinicians					
Quality of working life (eg, burnout, job satisfaction, engagement)			Not considered	Significant improvement of perceived well-being but still low values on a Likert scale (averaged of 0.5/5.0)	Survey to 99 control and 99 users on caregiver satisfaction. Slightly high scores in DAX^c^ users on engagement (3.62 vs 3.37), safety (4.16 vs 3.92), resilience/decompression (2.83 vs 2.81), and work-life balance (3.14 vs 2.90) and significant increase in after-hours work (4.69%)
Other outcomes for health care organizations					
Organizational performance			Time per notes per appointment decreased by 1.4 min on average and time after hours decreased by 7.4 min	Survey to 31 clinicians to assess the ease of completion of the documentation	Standard operational report on wRVU^d^ and no statistical differences in wRVU after adjusting panel size

a SSI: surgical site infection.

b FLOP: floating-point operations per second.

c DAX: Dragon Ambient Experience.

d wRVU: work relative value unit.

**Table 3.** Evaluation of included studies (continued from Table 2) using QUEST, Systems Engineering Initiative for Patient Safety (SEIPS) 3.0, and evaluation metrics proposed by Abbasian et al’s [26] frameworks.

Framework, dimensions, and components			Paper		
			Nguyen et al [33]	Owens et al [34]	Tierney et al [11]
Health care evaluation metric groups from Abbasian et al [26]					
Accuracy					
Intrinsic, SSI^a^, robustness, generalization, conciseness, up-to-dateness, groundedness			Reported the need to edit the notes sometimes for errors (groundedness)	Not considered	Not considered
Trustworthiness					
Safety and security, privacy, bias, interpretability			“Inserted odd things that only a computer could do” and “called the patient’s wife the adult female in the room” (interpretability)	Not considered	Embedded accuracy and bias in the overall score of the 35 notes evaluated (of 303,266 encounters using the tool). They reported an average rate of 4.6 out of 5.0 in the accurate domain and an average rate greater than 4.94 out of 5.0 in the free-from-bias domain
Empathy					
Emotional support, health literacy, fairness, personalization			Not considered	Not considered	Not considered
Performance					
Memory efficiency, FLOP^b^, token limit, number of parameters			Not considered	Not considered	Not considered
QUEST human evaluation framework					
Quality of information					
Accuracy			All participants reported the need to edit the notes, and “some edits were done to correct errors”	Not considered	Evaluated 35 notes out of 303,266 encounters using the tool. Accuracy evaluation was embedded in the overall score. The notes achieved an average rate of 4.6 in the accurate domain
Safety and harm					
Bias			Not considered	Not considered	Among the 35, bas evaluation was embedded in the overall score. The notes achieved an average rate greater than 4.94 out of 5.0 in the free-from-bias domain
Fabrication, falsification, or plagiarism			Some participants reported that the tool “inserted odd things that only a computer could do” and “called the patient’s wife the adult female in the room”	Not considered	Among the 35, transcripts averaged 48/50 points, with “few instances of hallucination” and “few instances in where the summary was missing some details”
SEIPS 3.0 Model					
Other outcomes for patients					
Physical, mental, and emotional health			Clinicians reported some patients expressed unease at having their visits recorded	Not considered	Not considered
Efficiency and effectiveness of care			Surveys and interviews on digital scribe characteristics. All participants reported the need to edit the notes, and “some edits were done to correct errors”	Not considered	Among the 35, transcripts averaged 48/50 points, with “few instances of hallucination” and “few instances in where the summary was missing some details”
Patient experience and satisfaction			Not considered	Not considered	21 patient surveys 71% reported they spent more time speaking with their physician, and 81% reported that their physician spent less time looking at the computer
Other outcomes for clinicians					
Quality of working life (eg, burnout, job satisfaction, engagement)			Mini *z* scores on clinician well-being No significant differences in burnout, work-related stressors, and sleep quality	Oldenburg Burnout Inventory Significant less burnout among users (mean 16.3 vs 18.4)	Not considered
Other outcomes for health care organizations					
Organizational performance			Surveys and interviews on digital scribe characteristics Variable time to feel comfortable with the tool, variable impact on documentation efficiency	Information on EPIC signal database Documentation time was reduced by 1.8 min on average, and time documenting outside work hours was reduced by 4 min	Electronic health record workload metrics Significant associations between the use of the tool and larger decreases in time after

a SSI: surgical site infection.

b FLOP: floating-point operations per second.

## Discussion

### Key Findings

Ambient scribe technology, a relatively recent advancement, is increasingly deployed to assist with clinical documentation, with the aim of reducing administrative burden on clinicians. In this rapid review, which screened 1450 studies, we identified 6 that assessed the real-world impact of AI-enabled scribes on documentation efficiency and related metrics. Findings showed consistent reductions in documentation time and modest reductions in “pajama time.” Improvements in clinician engagement were noted, although burnout levels remained unchanged, and patient satisfaction metrics showed no significant difference. Other key areas, including patient perceptions, cost-effectiveness, care quality, and safety concerns such as transcription errors and fabricated content, were infrequently addressed.

Across varied study designs and clinical settings, a consistent finding was a reduction in documentation time, with time savings ranging from 5.3 to 4.8 minutes per encounter in 1 study [11], and a decrease from 90.1 to 70.3 minutes per day in another [30]. Some studies also reported a modest reduction in “pajama time,” indicating a decline in after-hours documentation (from 14.9% to 14.2%) [32]. An increase in documentation length, with notes expanding by 30 to 50 words per entry or 542 characters in some cases [34], emerged as an unintended consequence. The effect of digital scribes on physician well-being was less consistent, with burnout scores (measured by Mini-Z or OLBI scales [34]) showing no improvement, although engagement scores, such as those measured by Press Ganey, were generally positive. Anecdotal feedback highlighted clinicians’ satisfaction with enhanced patient connection, with some describing AI scribes as a “game changer” [11]. From a productivity standpoint, digital scribes demonstrated cost savings over human scribes [30], although productivity gains were minimal [32]. While patient satisfaction metrics did not show significant differences, 1 study reported an improvement in patients’ perception of their experience [31]. A notable concern, albeit briefly discussed, was the potential for “hallucinations” [11]—where the AI may fabricate information, thereby altering the accuracy of medical documentation. Most studies fell short when compared to standardized frameworks (SEIPS 3.0, QUEST) in terms of completion.

These findings underscore the potential of AI-enabled scribes to reduce documentation burden and improve workflow efficiency, yet they raise important questions about the readiness of this technology for broad implementation. Although reduced documentation time is valuable in a health care landscape where documentation demands contribute significantly to clinician burnout, it remains uncertain whether these efficiency gains translate into more time for direct patient care or increased patient loads. This uncertainty highlights the need to evaluate how AI scribes influence patient-clinician interaction and, ultimately, patient care quality. Moreover, while shorter documentation times are beneficial, the increased note length may introduce redundancy, necessitating further study into the clinical relevance and user satisfaction with these longer, AI-generated notes. The perceived improvements in clinician engagement and work-life balance suggest that digital scribes may alleviate certain administrative burdens; however, they are unlikely to fully address the multifaceted issue of clinician burnout, which is influenced by factors such as workload, institutional culture, and support systems. Other key areas, including patient perceptions, cost-effectiveness, care quality, and safety concerns such as transcription errors and fabricated content, were infrequently addressed in the reviewed studies.

### Future Directions

Despite a growing interest in the use of ambient AI in health care, there is a dearth of high-quality, real-world evidence of its utility. In spite of a large number of studies (n=144) describing the use of digital scribes, many using the same technology, we found only 6 studies that spoke about real-world effectiveness. Among those 6 studies, the sample sizes were small, most papers had fewer than 100 participants, 1 described 12 physicians, and the other cited only anecdotal evidence. Future research on digital scribes should focus on large-scale, longitudinal studies to assess their long-term effectiveness, safety, and impact on clinician and patient outcomes. Standardized evaluation frameworks, such as QUEST and SEIPS 3.0, should be consistently applied to ensure reliable assessment of documentation quality, clinician well-being, and workflow integration. Furthermore, standardized burnout scales, such as the Maslach Burnout Inventory, could be considered while evaluating physician burnout [38]. In addition, patient-centered studies are needed to evaluate how these tools influence patient outcomes, satisfaction, and care quality. Economic analyses should be conducted to understand the cost-effectiveness of digital scribes, particularly in diverse health care settings. The financial implications of adopting digital scribes, including software costs, training, and potential productivity gains or losses, remain unclear. As health care systems increasingly invest in AI technologies, understanding their economic impact will be vital for informed decision-making. Data on the cost of implementation and current service pricing were opaque, both in the literature and in reviewing websites of individual vendors, with limited publicly facing pricing information making product comparison challenging. Finally, research should explore strategies to mitigate potential risks, such as transcription errors and “hallucinations,” to enhance trust and reliability in these emerging technologies. Overall, the focus should be on addressing the clinical relevance and long-term impact of AI-assisted documentation on both clinicians and patients.

### Limitations

This rapid review has several limitations. First, while the rapid review methodology enabled the timely synthesis of current evidence, it lacks the comprehensive rigor of a systematic review. This approach may have resulted in the omission of relevant studies, especially those in the gray literature or recently published works not yet indexed in major databases. Second, the small number of included studies and their heterogeneity in design, setting, and population limit the generalizability of the findings. Most studies were observational, with limited sample sizes and specific to certain clinical environments, such as dermatology and oncology. This variability restricts broader applicability across diverse health care settings. Third, the reliance on self-reported measures of documentation time and clinician well-being introduces potential biases, such as recall and social desirability bias. Objective metrics, such as direct observation or EHR log data, were rarely used, which could provide more reliable insights into the actual impact of digital scribes. Fourth, the included studies varied in their evaluation frameworks and outcome measures. There was a lack of consistent use of validated tools for assessing documentation quality, patient satisfaction, and clinician burnout, which hampers the ability to draw definitive conclusions about the effectiveness and safety of digital scribes. Finally, the rapidly evolving nature of digital scribe technology, driven by advancements in AI, means that the findings of this review may quickly become outdated. Several additional studies have been published since the completion of our review [38-45], further highlighting the importance of ongoing, rigorous evaluation of this rapidly evolving clinical technology. We recognize these contributions and note that our findings should be viewed as an early snapshot within a quickly expanding body of evidence. Continuous evaluation through robust, large-scale studies is essential to keep pace with technological developments and to provide more conclusive evidence on the long-term impact of digital scribes in clinical practice.

### Conclusions

Digital scribe technologies are being well received in their initial rollout, but it is important to note that there is still a severe paucity of data in real-world settings. Before further expansion is considered, robust large-scale studies on usability, acceptance, effectiveness, patient feedback, accuracy, safety, and cost should be conducted. Although the studies we reviewed reveal a positive trend, we hope that future larger, well-designed studies will comprehensively evaluate these tools and demonstrate that they indeed live up to their claimed promise in alleviating documentation burdens and associated concerns.

## Supplementary material

10.2196/76743 Multimedia Appendix 1 Full search strategy.

10.2196/76743 Checklist 1 PRISMA checklist.

## Abbreviations

AI: artificial intelligence
DAX: Dragon Ambient Experience
EHR: electronic health record
OLBI: Oldenburg Burnout Inventory
PABAK: prevalence-adjusted bias-adjusted κ
PRISMA: Preferred Reporting Items for Systematic Reviews and Meta-Analyses
SEIPS: Systems Engineering Initiative for Patient Safety
