# Correction: Real-World Evidence Synthesis of Digital Scribes Using Ambient Listening and Generative Artificial Intelligence for Clinician Documentation Workflows: Rapid Review

**DOI:** 10.2196/93250

**Published:** 2026-03-13

**Authors:** Naga Sasidhar Kanaparthy, Yenny Villuendas-Rey, Tolulope Bakare, Zihan Diao, Mark Iscoe, Andrew Loza, Donald Wright, Conrad Safranek, Isaac V Faustino, Alexandria Brackett, Edward R Melnick, R Andrew Taylor

**Affiliations:** 1 Department of Biomedical Informatics and Data Science, Yale School of Medicine, New Haven, CT, United States; 2 VA Connecticut Healthcare System, US Department of Veterans Affairs, West Haven, CT, United States; 3 Department of Emergency Medicine, Yale School of Medicine, 464 Congress Avenue, #260, New Haven, CT, 06519, United States, 1 203-737-7694; 4 Centro de Innovación y Desarrollo Tecnológico en Cómputo CIDETEC, Instituto Politécnico Nacional, Mexico City, Mexico; 5 Department of Biostatistics (Health Informatics Division), Yale School of Public Health, New Haven, CT, United States; 6 Harvey Cushing/John Hay Whitney Medical Library, Yale University, New Haven, CT, United States; 7 Department of Emergency Medicine, University of Virginia, Charlottesville, VA, United States

 In “Real-World Evidence Synthesis of Digital Scribes Using Ambient Listening and Generative Artificial Intelligence for Clinician Documentation Workflows: Rapid Review” [1], the authors noted one omission.

In addition to the funding already acknowledged, the authors would like to add the following:

MI’s work on this publication was made possible by CTSA Grant Number KL2 TR001862 from the National Center for Advancing Translational Science, a component of the National Institutes of Health.

The correction will appear in the online version of the paper on the JMIR Publications website, together with the publication of this correction notice. Because this was made after submission to PubMed, PubMed Central, and other full-text repositories, the corrected article has also been resubmitted to those repositories.

## References

[1] Kanaparthy NS, Villuendas-Rey Y, Bakare T (2025). Real-world evidence synthesis of digital scribes using ambient listening and generative artificial intelligence for clinician documentation workflows: rapid review. JMIR AI.

