# Supplementary material for: Real-World Evidence Synthesis of Digital Scribes Using Ambient Listening and Generative Artificial Intelligence for Clinician Documentation Workflows: Rapid Review
Source: JMIR AI. 2025 Oct 10;4:e76743. doi: 10.2196/76743 (PMC12513689; doi:10.2196/76743)
Supplement: Multimedia Appendix 1 [file ai-v4-e76743-s001.docx]

### Supplement 1 : Complete Search Strategy

*Ovid MEDLINE(R) ALL <1946 to July 30, 2024>*

*1 Speech Recognition Software/ 842*

*2 ((ambient* or digital* or model* or audio*) adj5 (scribe* or transcribe* or transcrip* or voice technolog* or listen* or dictation*)).mp. 24378*

*3 1 or 2 25201*

*4 exp Medical Records/ 162383*

*5 Patient Discharge Summaries/ 293*

*6 exp Documentation/ 966749*

*7 ((medical or medicine or health or clinical or patient*) adj2 (record* or document* or discharge summar* or note*)).mp. 392346*

*8 4 or 5 or 6 or 7 1388495*

*9 3 and 8 2019*

*10 limit 9 to yr="2014 - 2024" 789*

*Embase <1974 to 2024 July 30>*

*1 exp automatic speech recognition/ 1443*

*2 ((ambient* or digital* or model* or audio*) adj5 (scribe* or transcribe* or transcrip* or voice technolog* or listen* or dictation*)).mp. 34186*

*3 1 or 2 35583*

*4 exp medical record/ 353478*

*5 medical documentation/ 31353*

*6 ((medical or medicine or health or clinical or patient*) adj2 (record* or document* or discharge summar* or note*)).mp. 813034*

*7 4 or 5 or 6 813483*

*8 3 and 7 1021*

*9 limit 8 to yr="2014 - 2024" 734*

*# Web of Science*

*# Entitlements:*

*- WOS.IC: 1993 to 2024*

*- WOS.CCR: 1985 to 2024*

*- WOS.SCI: 1900 to 2024*

*- WOS.AHCI: 1975 to 2024*

*- WOS.BHCI: 2005 to 2024*

*- WOS.BSCI: 2005 to 2024*

*- WOS.ESCI: 2019 to 2024*

*- WOS.ISTP: 1991 to 2024*

*- WOS.SSCI: 1900 to 2024*

*- WOS.ISSHP: 1991 to 2024*

*# Searches:*

*1: TS=((ambient* or digital* or model* or audio*) NEAR/4 (scribe* or transcribe* or transcrip* or "voice technolog*" or listen* or dictation*)) Results: 27729*

*2: TS=((medical or medicine or health or clinical or patient*) NEAR/1 (record* or document* or "discharge summar*" or note*)) Results: 290431*

*3: #2 AND #1 Results: 353*

*4: #2 AND #1 Timespan: 2014-01-01 to 2024-12-31 Results: 291*

*Cochrane (CENTRAl/Trials & Cochrane reviews)*

*ID Search Hits*

*#1 (ambient* or digital* or model* or audio*) NEAR/4 (scribe* or transcribe* or transcrip* or voice technolog* or listen* or dictation*) 3035*

*#2 (medical or medicine or health or clinical or patient*) NEAR/1 (record* or document* or discharge summary or note*) 34251*

*#3 #1 AND #2 with Publication Year from 2014 to 2024, in Trials 137*

*#4 #1 AND #2 with Cochrane Library publication date Between Jan 2014 and Dec 2024, in Cochrane Reviews 37*

*#5 #3 OR #4 174*

*PubMed Central, 192 results*

*(((((((ambient scribe*[Body - Key Terms] OR ambient transcribe*[Body - Key Terms] OR ambient transcrip*[Body - Key Terms] OR ambient voice technolog*[Body - Key Terms] OR ambient listen*[Body - Key Terms] OR ambient dictation*[Body - Key Terms])) OR (digital* scribe*[Body - Key Terms] OR digital* transcribe*[Body - Key Terms] OR digital* transcrip*[Body - Key Terms] OR digital* voice technolog*[Body - Key Terms] OR digital* listen*[Body - Key Terms] OR digital* dictation*[Body - Key Terms])) OR (model* scribe*[Body - Key Terms] OR model* transcribe*[Body - Key Terms] OR model* transcrip*[Body - Key Terms] OR model* voice technolog*[Body - Key Terms] OR model* listen*[Body - Key Terms] OR model* dictation*[Body - Key Terms])) OR (audio* scribe*[Body - Key Terms] OR audio* transcribe*[Body - Key Terms] OR audio* transcrip*[Body - Key Terms] OR audio* voice technolog*[Body - Key Terms] OR audio* listen*[Body - Key Terms] OR audio* dictation*[Body - Key Terms])))) AND ((((((medical record*[Body - Key Terms] OR medical document*[Body - Key Terms] OR medical discharge summary[Body - Key Terms] OR medical note*[Body - Key Terms])) OR (medicine record*[Body - Key Terms] OR medicine document*[Body - Key Terms] OR medicine discharge summary[Body - Key Terms] OR medicine note*[Body - Key Terms])) OR (health record*[Body - Key Terms] OR health document*[Body - Key Terms] OR health discharge summary[Body - Key Terms] OR health note*[Body - Key Terms])) OR (clinical record*[Body - Key Terms] OR clinical document*[Body - Key Terms] OR clinical discharge summary[Body - Key Terms] OR clinical note*[Body - Key Terms])) OR (patient* record*[Body - Key Terms] OR patient* document*[Body - Key Terms] OR patient* discharge summary[Body - Key Terms] OR patient* note*[Body - Key Terms])) Filters: Publication date from 2014/01/01 to 2024/12/31*
